# ORAI Ca^2+^ Channels in Cancers and Therapeutic Interventions

**DOI:** 10.3390/biom14040417

**Published:** 2024-03-29

**Authors:** Qian Zhang, Chen Wang, Lian He

**Affiliations:** Department of Pharmacology, Joint Laboratory of Guangdong–Hong Kong Universities for Vascular Homeostasis and Diseases, School of Medicine, Southern University of Science and Technology, Shenzhen 518055, China; zhangq8@sustech.edu.cn (Q.Z.); lnaswc@foxmail.com (C.W.)

**Keywords:** CRAC channels, store-operated Ca^2+^ entry, ORAI, optogenetics, cancers

## Abstract

The ORAI proteins serve as crucial pore-forming subunits of calcium-release-activated calcium (CRAC) channels, pivotal in regulating downstream calcium-related signaling pathways. Dysregulated calcium homeostasis arising from mutations and post-translational modifications in ORAI can lead to immune disorders, myopathy, cardiovascular diseases, and even cancers. Small molecules targeting ORAI present an approach for calcium signaling modulation. Moreover, emerging techniques like optogenetics and optochemistry aim to offer more precise regulation of ORAI. This review focuses on the role of ORAI in cancers, providing a concise overview of their significance in the initiation and progression of cancers. Additionally, it highlights state-of-the-art techniques for ORAI channel modulation, including advanced optical tools, potent pharmacological inhibitors, and antibodies. These novel strategies offer promising avenues for the functional regulation of ORAI in research and may inspire innovative approaches to cancer therapy targeting ORAI.

## 1. Introduction

The majority of cellular activities, including but not limited to cell proliferation [1], migration [2], transformation [3], and mitophagy [4], utilize Ca^2+^ as a second messenger. Dysfunction of Ca^2+^ regulation can lead to various diseases, especially cancers. In non-excitable cells, store-operated Ca^2+^ entry (SOCE) is one of the most common routes mediating Ca^2+^ influx. It is triggered by the depletion of intracellular Ca^2+^ stores and subsequently activates stromal interaction molecule (STIM), an endoplasmic reticulum (ER)-resident Ca^2+^ sensor. STIM then gates and opens cell-membrane-localized ORAI Ca^2+^ channels [5].

**Figure 1:**
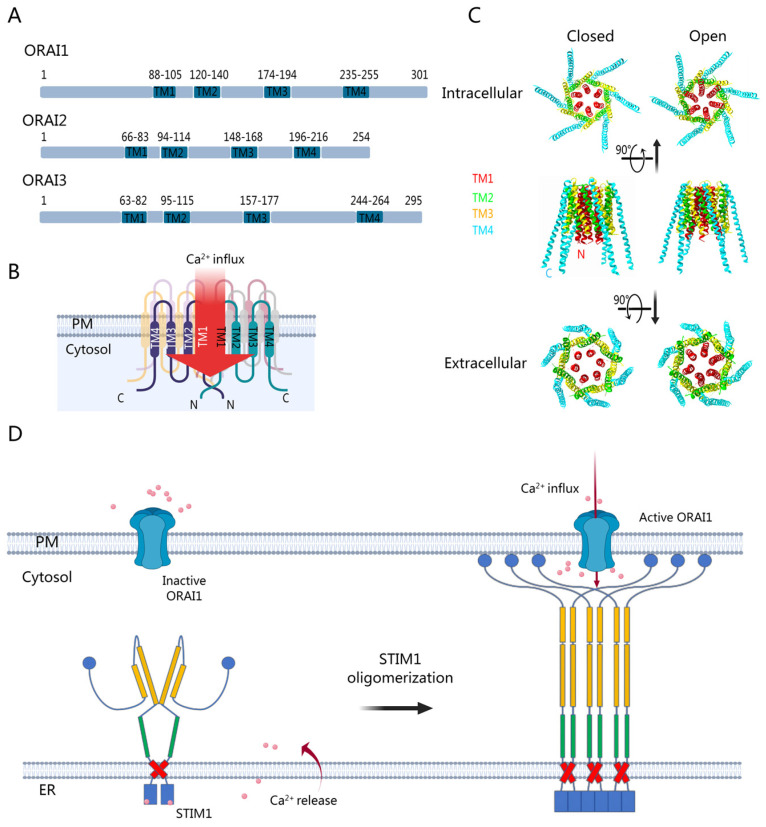
Structures and activation of human ORAI proteins. (**A**) The ORAI family consists of three highly conserved homologous proteins, named ORAI1 to ORAI3. They are tetra-spanning plasma membrane proteins. Besides the N-terminus and C-terminus, four transmembrane domains (TMs) and three connecting loops are alternately arranged. (**B**) Diagram illustrating the hexameric ORAI channel. TM1 helices in the innermost layer form the inner wall of the channel, while the other TM helices are orderly arranged from inside to outside. (**C**) The 3D structures of dOrai in the closed state (PBD ID: 6BBH) and open state (PBD ID: 6BBF) shown from intracellular, side, and extracellular views, separately. All images were created by ChimeraX. (**D**) Activation model of the CRAC channel. Depletion of ER luminal Ca^2+^ induces STIM1 oligomerization and interaction with ORAI1, sequentially causing Ca^2+^ entry. PM: plasma membrane.

The ORAI family consists of three highly conserved homologous subtypes: ORAI1, ORAI2, and ORAI3 (Figure 1A). ORAI1 and ORAI2 in vertebrates evolved from the single ORAI protein in invertebrates, and the duplication of the *Orai1* gene led to ORAI3 in mammals. They are uniformly distributed in the plasma membrane, but the function of ORAI1 is much better known compared to the other two. Each ORAI protein has four transmembrane domains (TM1–4) connected by one intracellular and two extracellular loops, with both N- and C-termini facing the cytoplasm (Figure 1B). They are all involved in store-operated STIM1-mediated activation with high Ca^2+^ selectivity, yet they possess distinct functional and structural characteristics. While the transmembrane domains in ORAI homologs exhibit approximately 80% sequence identity, notable variations in sequence are evident within the cytosolic and extracellular domains, leading to diverse functional alterations in ORAI channels [6,7]. ORAI3 and ORAI2 play crucial roles in mediating low-range and mid-range Ca^2+^ oscillatory responses, while ORAI1 mediates high-range plateaus. ORAI1, in particular, is distinguished by a lengthy N-terminus containing a polybasic- and proline-rich region, which likely contributes to its ability to induce 2–3-fold maximum currents compared with ORAI2 and ORAI3 [8]. Variations in the cytosolic regions of ORAI channels result in different Ca^2+^-dependent inactivation patterns [6,9]. Moreover, the three isoforms exhibit distinct pharmacological profiles [10]. Despite this, the ORAI isoforms are interrelated. While ORAI1 serves as a crucial subunit of CRAC channels, accumulating evidence suggests that ORAI2 and ORAI3 may heteromerize with ORAI1 to form native CRAC channels, thereby negatively regulating the function of ORAI1 [11,12]. For a long time after its first discovery in 2006 [13], the human ORAI channel was believed to be a tetramer [14]. However, in 2012, the crystal structure of the *Drosophila melanogaster* Orai (dOrai) revealed a hexameric structure [15], with these two species sharing 73% sequence identity in TM regions [16]. Undoubtedly, there is strong evidence indicating that the human ORAI1 channel also functions as a hexamer [17,18]. From the studies of dOrai, it is well established that six TM1 units form the inner wall of the ORAI channel, while the other three TMs are sequentially layered from inside to outside, with TM4 on the outermost side (Figure 1B,C). Given that ORAI is a key component of the Ca^2+^ release-activated Ca^2+^ (CRAC) channel, abnormal alterations in ORAI would lead to Ca^2+^ dysregulation and subsequentially cause various pathological conditions, including immune diseases [13], cardiovascular diseases [19], as well as cancers [20,21,22]. In the present work, we aim to elucidate the activation and modulation mechanism of the ORAI channel, as well as to summarize the latest research on ORAI-related diseases.

## 2. ORAI Activation and Modulation

### 2.1. The Mechanism of ORAI Channel Activation and Inactivation

The activation of CRAC channels begins with ER-stored Ca^2+^ depletion, mediated by the activation of G protein-coupled receptors or receptor tyrosine kinases. The dissociation of Ca^2+^ from the EF-hand domain results in a conformational rearrangement of STIM and the release of the STIM–ORAI activating region (SOAR), which directly gates ORAI1. The binding motif in ORAI was initially controversial. The N-terminal, but not the C-terminal, was thought to bind with STIM1 due to its proximity to the pore-lining TM1. However, more recent studies have identified that TM4 with cytoplasmic C-terminal has a higher binding affinity [23,24]. By comparing the closed and open states of dOrai, Liu et al. found that all six TM4 regions are fully extended and rotate clockwise, subsequentially causing N-termini to twist outward in a counterclockwise manner during activation [25,26]. Interestingly, the positively charged region in the basic section of the TM1 helix is critical for Ca^2+^ permeation by recruiting anions and thus increasing the potential gradient across the plasma membrane [25]. Mutations in the positively charged amino acids lead to dysfunctional ORAI channels, as shown in Table 1. Under normal conditions, Ca^2+^ flow into the cytosol stops when reaching a charge balance, owing to the fast Ca^2+^-dependent inactivation (CDI) of the CRAC channel. STIM1 was previously thought to be essential for CDI until Yeung et al. found that gain-of-function (GOF) mutations in L138 and T92 presented CDI in the absence of STIM1 [27]. The truncation of the ORAI1 C-terminal end abolished the mutation-induced CDI, indicating that the C-terminus is not only involved in ORAI channel opening but also closing. TM2 and TM3 do not show significant conformational differences between open and closed states of the ORAI channel. However, this does not mean that they are not important for ORAI-mediated Ca^2+^ influx. Tiffner et al. published a study in which multiple paired mutations in all TMs were introduced to assess the checkpoints of ORAI1 pore opening [28].

Without question, any minor changes in protein sequence, such as mutations or post-translational modifications (PTMs), have the potential to affect ORAI channel function. Persistent activation or dysfunction of the ORAI channel can lead to pathological states. Next, we would like to emphasize the sequence changes and post-translational modifications in ORAI, particularly ORAI1, that are associated with diseases. These may provide valuable insights for further exploration into potential disease treatments.

### 2.2. Mutations and Post-Translational Modifications of ORAI in Diseases

ORAI mutations, many of which were located in TM1, were primarily associated with immune diseases and were reviewed in 2010 [46]. However, as research progressed, more mutation sites and details have been uncovered. Generally, loss-of-function (LOF) mutations lead to insufficient cellular activities and diseases such as immunodeficiency, ectodermal dysplasia, and muscular hypotonia [13,31,47,48]. Conversely, gain-of-function mutations cause extensive Ca^2+^ influx and cellular dysfunctions, including miosis, tubular myopathy [27,33,49], and cancers [41,45]. In recent years, Yu et al. reported a case of a 22-month-old female patient with combined immunodeficiency, muscular hypotonia, and anhidrotic ectodermal dysplasia due to a homozygous C126R mutation in the TM2 of ORAI1 [37]. C126R ORAI1 was localized and retained in the ER but not PM; thus, it was unable to properly form CRAC channels. Meanwhile, the mutation of L138F in TM2 evokes constitutive channel activation in the absence of STIM1 [27], which is associated with tubular aggregate myopathy (TAM). More disease-related mutations in ORAI1 are summarized in Table 1.

Another method that affects the function of the ORAI channel without altering the protein sequence is post-translational modification, including phosphorylation, glycosylation, acylation, and redox modulation [50]. Although the former three are the most universal PTMs in controlling cellular signal pathways [51], there are not many reported diseases directly related to ORAI PTMs. Theoretically, all the intracellular regions of ORAI1 are potential phosphorylation sites by protein kinase C (PKC). Ser-27 and Ser-30 in the N-terminus have been proven to be key regulatory points [52], inducing loss-of-function of the ORAI channel in invasive melanoma when phosphorylated [53]. Unlike phosphorylation, the glycosylation site in ORAI1 only exists in the extracellular loop3 at Asn–233, and the eventual effects depend on cell types [54,55]. It has been found that glycosylation at this site restores the SOCE in fibroblasts from SCID patients caused by the N233A mutation [43]. As for acetylation, C143 is the only acting site in ORAI1, improving STIM1–ORAI1 interaction and channel activation [56]. Compared to numerous reported PTMs in STIM1, little is known about the effects of ORAI PTMs on human diseases. More studies are needed to explore the further mechanisms. 

### 2.3. Techniques to Modulate ORAI Channel Function

Given the significant impact of calcium channels on various physiological processes, precise control is essential for both fundamental research and clinical applications. Here, several techniques are provided as outlined below.

#### 2.3.1. Optical Tools in ORAI Regulation

Relying on the rapid development of optogenetics, reversible and spatiotemporal control of calcium entry becomes feasible. The conformations of photoswitchable proteins are altered under light illumination, enabling their widespread use in the functional control of proteins of interest (POIs) through optogenetic oligomerization [57] or photo-caging [58]. The stimulation spectrum for various photosensors spans a wide range of light, from ultraviolet (UV) to near-infrared (NIR) wavelengths [58]. Currently, most studies involving optogenetic CRAC modulation focus on STIM1 [59,60,61,62,63]. Only a few labs have utilized ORAI1 as the POI, which can activate calcium entry independent of STIM1. 

**Figure 2:**
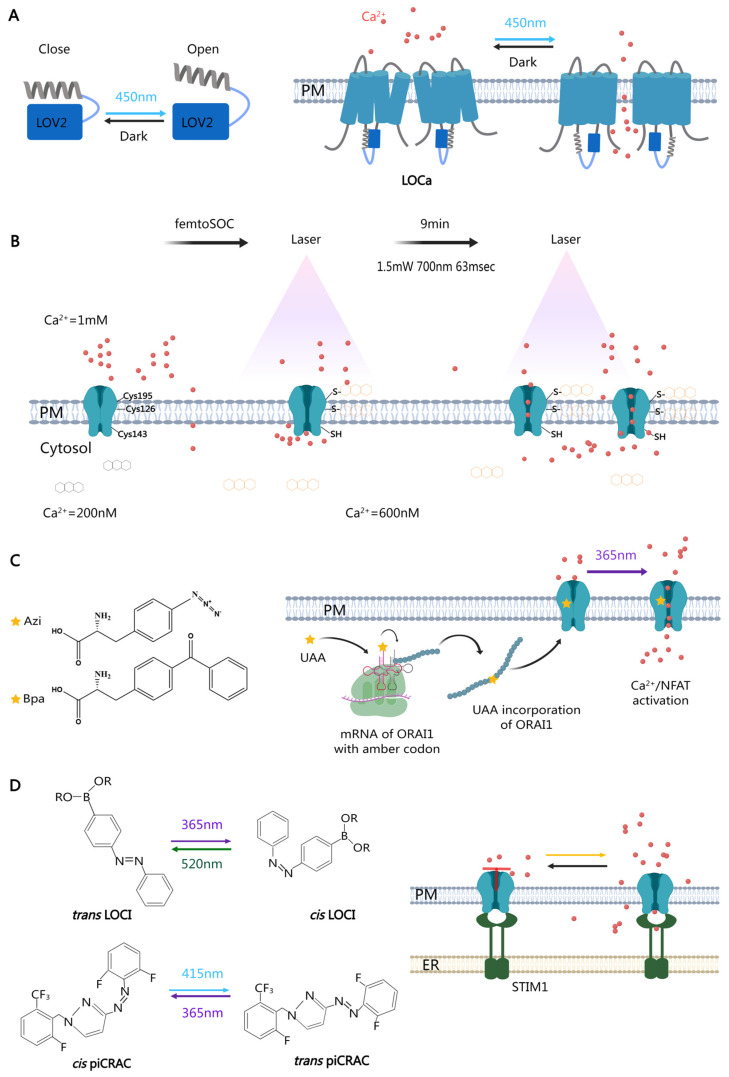
Optical methods for ORAI1 channel modulating. (**A**) Directly interfering with ORAI1 channels by blue light. By introducing mutations and inserting the LOV2 domain between TM2 and TM3, a light-operated calcium channel (LOCa) was generated, inducing calcium entry upon blue light illumination. (**B**) Schematic diagram showing the activation mechanism of femtoSOC. With the stimulation of femtosecond laser pulses, photoexcited flavins are linked to cysteine residues in ORAI1 via thioether bonds, thereby inducing ORAI1 channel opening and calcium influx. (**C**) Graphical illustration of photocrosslinking-induced ORAI1 activation. The photocrosslinking unnatural amino acids (UAAs), p-azido-L-phenylalanine (Azi) and p-benzoyl-L-phenylalanine (Bpa), were introduced into the ORAI1 peptide chain through genetic code expansion technology. These UAAs respond to UV light illumination and control the state of ORAI1 channels. (**D**) The photochemical regulation of ORAI1 channels. The small-molecule inhibitor 2-aminoethoxydiphenyl borate (2-APB) was fused with the photoswitching molecule azobenzene to create light-operated CRAC channel inhibitors (LOCIs). CRAC small-molecule inhibitors were fused with azopyrazole to develop photoswitchable CRAC channel inhibitors (piCRACs). Both LOCI and piCRAC affect the activation of ORAI channels. PM: plasma membrane.

He et al. designed a light-operated ORAI channel (LOCa) utilizing the light-oxygen-voltage-sensing domain 2 (LOV2) from *Avena sativa* phototropin 1 [64] (Figure 2A). By inserting LOV2 into various regions of ORAI1 and introducing random mutations into the ORAI1 sequence, they generated a series of blue-light-gated calcium channels capable of reversibly activating calcium entry without exogenous cofactors. After rounds of screening, the constitutively active ORAI1 induced by H171D/P245T mutations, with the insertion of LOV2 between TM2 and TM3, was named LOCa3. With blue light illumination, the Jα helix of LOV2 loosened, releasing the ORAI channel from its caging. LOCa was successfully applied to activate transcription, increase cell renewal in vitro, and alleviate neurodegeneration in vivo. Thus, LOCa is presented as a single-component, photoswitchable Ca^2+^-selective channel amenable to many biotechnological and biomedical applications. Further exploration of photoswitchable protein-fused ORAI channels is warranted to provide more options in the future.

Cheng et al. devised a system femtoSOC for direct control of ORAI1 channels solely by ultrafast laser, eliminating the need for optogenetic tools or any other exogenous reagents [65] (Figure 2B). Photoexcited flavins were introduced to covalently bind cysteine residues in ORAI1 via thioether bonds, thereby facilitating ORAI1 channel opening independently of STIM1. Using femtosecond laser pulses (1.5 mW, 700 nm, 63 ms), a 2 × 2 µm^2^ area of the plasma membrane was scanned, and flavins in the region were photoexcited, subsequently binding to Cys126 and Cys195 residues in ORAI1, allowing extracellular Ca^2+^ to enter the cell. With prolonged illumination, ORAI1 channels in the surrounding area moved and aggregated in the femtoSOC-focused area. The system successfully induced downstream signaling pathways in Hela cells and activated neurons in the mouse brain. 

Even without the integration of extra components, there are still methods available to control CRAC channels using light. The emerging optoproteomics technology provides an alternative strategy to confer light sensitivity not only to POIs but also to individual amino acids [66,67]. Maltan et al. utilized photocrosslinking unnatural amino acids (UAAs) to achieve temporally precise, light-mediated remote control over ORAI1 channel activation at the level of single amino acids [68] (Figure 2C). Employing genetic code expansion (GCE) technology [69], they incorporated unnatural amino acids such as p-azido-L-phenylalanine (Azi) and p-benzoyl-L-phenylalanine (Bpa) at critical checkpoints in ORAI1. Upon irradiation with UV light (365 nm), these photocrosslinking UAAs become reactive and form covalent bonds with nearby residues, enabling CRAC channel activation [70]. Finally, A137Bpa mutation in Orai1 TM2, L174Bpa in TM3, and A254Azi inTM4 were selected for their robust UV light-induced activation. The results also demonstrated that these mutations successfully initiated the downstream signaling pathways after calcium entry. Direct interference with ORAI conformation appears more challenging than with STIM1, as the former is a multi-transmembrane protein with various activation checkpoints and firm localization. So far, the optical control methods for ORAI remain limited, not just in quantity but also in practical use. 

#### 2.3.2. Novel Pharmacological Inhibitors for the ORAI Channel

The inhibitors of the CRAC channel either target the pore of ORAI or interfere with STIM–ORAI interaction, as reviewed [71,72,73]. Small-molecule compounds for ORAI inhibition have been extensively developed, with some of them undergoing clinical trials [74,75]. For instance, CM4620 has progressed to clinical trials for treating patients with severe COVID-19 pneumonia [76]. Here, we would like to present up-to-date research on novel compounds or new applications of canonical inhibitors. Most of these compounds target more than one channel component, meaning they often have off-target effects. Recently, Ahmad et al. developed an ORAI1-specific inhibitor, ELD607, which exhibits high inhibitory efficiency (IC_50_ = 9 nM in HEK293T cells) and reduces neutrophilia and lung bacteria in vivo through macrophage-mediated resolution [77]. Kong et al. identified two novel and selective CRAC channel inhibitors that directly act on the ORAI1 protein [78]. One is an indole-like compound, C63368, and the other is pyrazole core-containing compound C79413, both potently and reversibly inhibiting the CRAC channel with low micromolar IC50s while sparing various off-target ion channels. In vivo studies have shown great therapeutic benefits in psoriasis and colitis animal models of autoimmune disorders. Interestingly, Yuan et al. reported a natural compound, celastrol, as a SOCE inhibitor, affecting both ORAI1 and STIM1 [79]. 

While some scientists explore new modifications for existing inhibitors, the combination of small compounds with optogenetic tools has made it possible to precisely manipulate endogenous CRAC channels in a spatiotemporal manner. Udasin et al. reported the synthesis of azoboronate light-operated CRAC channel inhibitors (LOCIs) that allowed for dynamic and fully reversible remote modulation of native CRAC channel function using ultraviolet (UV) and visible light [80] (Figure 2D). The small-molecule azobenzene, which switches conformation upon illumination at the wavelengths of 365 nm and 520 nm, was fused to pharmacological analogs of the CRAC channel inhibitor 2-aminoethoxydiphenyl borate (2-APB). Unlike 2-APB, LOCI directly affected ORAI1 and ORAI3 at an extracellular-facing site, rather than intervening in the STIM1–ORAI1 interaction, and blocked Ca^2+^ entry in Jurkat T cells and a metastatic breast cancer mouse model. UV light induced the trans-to-cis photoisomerization of LOCI, disabling the inhibition and subsequently inducing Ca^2+^ entry. 

Yang et al. published a similar paper earlier in 2020 [81]. They presented azopyrazole-derived photoswitchable CRAC channel inhibitors (piCRACs), which enable optical inhibition of store-operated Ca^2+^ influx and downstream signaling (Figure 2D). Unlike azoboronate, the conformation of azopyrazole switches between UV light (365 nm) and blue light (415 nm). The CRAC inhibitors GSK–5498A, GSK–7975A, GSK–5503A, and Synta 66 were chosen to construct piCRACs. piCRAC–1 has been applied in vivo to alleviate thrombocytopenia and hemorrhage in a zebrafish model of Stormorken syndrome in a light-dependent manner.

In a recently published work, Tscherrig et al. synthesized several novel small-molecule probes based on the known SOCE inhibitor GSK–7975A [82]. These probes incorporated various functionalities, including photo-caging, photocrosslinking, biotin, clickable moieties, and deuterium labels. Remarkably, most of these compounds retained inhibitory activity. Undoubtedly, this research underscores the versatility of modifications achievable within the parent ligand scaffold.

#### 2.3.3. ORAI1-Targeted Antibodies

Theoretically, ORAI proteins offer multiple potential target sites for antibodies. However, only a limited number of antibodies against ORAI1 have been reported in living cells or organs. Early in 2008, Jardin et al. electrotransjected an anti-ORAI1 antibody, directed toward the amino acid sequence 288–301 of the human ORAI1 C-terminal region involved in the interaction with STIM1, into platelets to successfully inhibit SOCE [83]. In 2013, Lin et al. [84] and Cox et al. [85] reported monoclonal antibodies against loop2 of ORAI1, separately, effectively abrogating the SOCE activity in Jurkat T cells as well as HEK293T cells. Subsequently, in 2015, a study utilized a human anti-ORAI1 monoclonal antibody to inhibit CRAC channels, leading to reduced T-cell-derived cytokine production. However, this antibody failed to inhibit a T-cell-dependent antibody response in cynomolgus monkeys [86]. Despite these advancements, there is a notable absence of recent studies on the development of anti-ORAI1 monoclonal antibodies, including nanobodies. Nanobodies, also known as single-domain-based VHHs, are antibody fragments derived from *Camelidae* heavy-chain-only IgG antibodies. These nanobodies offer several advantages, including their small size, high binding affinity, exceptional specificity, minimal off-target effects, and excellent stability in extreme conditions. Given their remarkable properties, nanobodies warrant further exploration and broader investigation across diverse fields, including basic research, diagnostics, and therapeutics.

## 3. ORAI and Cancers

Dysfunction of CRAC channels can contribute to a myriad of diseases, including bronchial asthma [87,88], airway hyperreactivity [89], cardiovascular diseases [90,91,92], hypocalcemia [93], brain diseases [94,95,96], gastroenteric inflammation [97], as well as various carcinomas [98,99,100,101,102]. Here, we will focus on the role of ORAI in cancer.

### 3.1. ORAI Expression and Its Role in Various Cancers

According to an estimate from the World Health Organization (https://gco.iarc.fr/tomorrow, accessed on 28 December 2023), approximately 30.2 million people are projected to be affected by cancer in 2040. Studies have reported that ORAI1 is highly expressed in nearly 40 types of carcinomas [99,103]. 

Prostate cancer (PCa) stands as the most prevalent non-cutaneous tumor in males worldwide. All three subtypes of ORAI proteins exhibit expression in both normal and cancerous epithelial prostatic cells. Distinct isoforms of the ORAI channel correlate with diverse outcomes of PCa. Notably, the overexpression of ORAI3, and not only ORAI1 [104], assumes a pivotal role in PCa regulation [101,105]. In an aged knock-in mouse prostate adenocarcinoma model, ORAI3 and STIM2 mRNA levels were significantly increased compared to ORAI1 and STIM1, and ORAI3–STIM2 interaction was detected in PC–3 cells under basal conditions [101]. This ORAI3–STIM2 complex enhances PCa cells’ progression through evading mitotic catastrophe. Moreover, ORAI3 and ORAI1 can form heteromultimeric channels that induce store-independent Ca^2+^ entry (SICE) activation by leukotriene C4 (LTC4) or arachidonic acid, known as LTC4-regulated Ca^2+^ (LRC) channels and arachidonic-acid-regulated (ARC) Ca^2+^ channels, respectively [106]. Evidence suggests that ORAI3 governs PCa by creating either ARC channels or LRC channels, even through ORAI3-encoded SOCs in malignant cells instead of healthy cells, which controls cancer hallmarks and promotes carcinogenesis [107]. Interestingly, ORAI2 overexpression correlates with a reduced risk of systemic recurrence following radical prostatectomy [108]. On the other hand, the downregulation of ORAI1 and ORAI3 promotes cell proliferation and decreases apoptosis rates [109,110]. ORAI1 mediates PCa cell apoptosis in response to chemotherapeutics such as cisplatin and oxaliplatin. Notably, in steroid-deprived prostate cancer cells, ORAI1 downregulation confers resistance to cisplatin-induced apoptosis [110,111].

Similar to PCa, the expression of ORAI3 channels is often increased in breast cancer (BC) [105,112,113], involving cancer cell proliferation, cell cycle development, and survival. Hasna et al. found that the overexpression of ORAI3 in BC led to a decrease in cell mortality and apoptosis, and an increase in resistance to apoptosis inducers and chemotherapeutic drugs through downregulation of the p53 tumor suppressor protein expression that was mediated by the pro-survival PI3K/Sgk–1/Sek–1 pathway [114]. While ORAI1 is known to be highly expressed in breast cancers, it was only recently revealed that both the full-length subtype Orai1α and the truncated subtype Orai1β, lacking the N-terminal 63 amino acids, support SOCE in triple-negative MDA–MB–231-derived breast cancer stem cells with similar efficiency, as well as cyclooxygenase (COX) activation and mammosphere formation [115].

ORAI1 expression was found to be elevated in gastric cancer (GC) tissues compared to adjacent non-tumor tissues. In a study involving 327 GC patients, higher Orai1 expression was correlated with advanced disease stages, more frequent recurrence, and increased mortality rates [116]. Knockdown of ORAI1 resulted in reduced proliferation, migration, and invasion of two gastric cancer cell lines. Knockdown of ORAI3 significantly reduced SOCE and inhibited proliferation by arresting non-small-cell lung cancer cell lines in the G0/G1 phase [117]. Elevated expression of ORAI1 in human oral cancer led to sustained Ca^2+^ influx. Son et al. pointed out that ORAI1 regulated many genes encoding oral cancer markers, including metalloproteases (MMPs) and pain modulators, through RNA-Seq analysis [118]. Lack of ORAI1 resulted in smaller oral cancer tumors and reduced MMP1 expression, which in turn diminished the activation of action potentials in trigeminal ganglia neurons.

**Figure 3:**
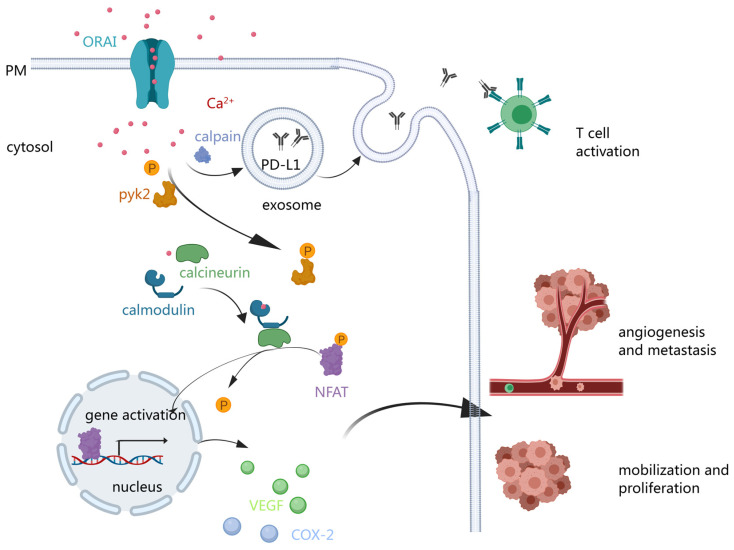
The role of the ORAI calcium channel in the initiation and development of tumors. The influx of Ca^2+^ resulting from ORAI channel activation stimulates the calcineurin/NFAT pathway, leading to the transcription of various cytokines that enhance cancer proliferation and metastasis. Furthermore, Ca^2+^ facilitates the excretion of PD–L1 via exosomes, activating T cells.

The activation of ORAI1 typically triggers calcineurin activation and subsequent nuclear factor of activated T cells (NFAT) translocation to the nucleus [119,120], involved in cell proliferation [121,122,123,124], apoptosis resistance [124], angiogenesis, cell invasion, migration [121,123,125], and metastasis [102,117,126] (Figure 3). The downstream signaling pathways encompass basal Ca^2+^ signaling [123], ERK (the extracellular signal-regulated kinase) signaling pathway, AKT/mTOR signaling pathway, focal adhesion turnover [127], FAK (focal adhesion kinase) tyrosine phosphorylation, NFAT signaling, and interleukin-6 (IL-6) signaling [122], among others. Next, we will delve into how ORAI contributes to the process of tumorigenesis. 

### 3.2. ORAI-Mediated Cancer Cell Epithelial-to-Mesenchymal Transition and Invasion

Epithelial–mesenchymal transition (EMT) is a cellular process through which epithelial characteristics transition to mesenchymal phenotypes, resulting in functional changes such as enhanced cell migration and invasion, typically induced by signals from the microenvironment [128]. EMT involves the loss of stable epithelial cell–cell junctions, apical–basal polarity, and interactions with the basement membrane. This process is a crucial program in cancer progression [129]. Remodeling of calcium signaling via SOCE actively contributes to EMT, with amplified ORAI1 expression in cancers promoting this transition. For instance, ORAI1 has been shown to enhance gastric cancer cell migration and invasion by targeting MACC1 (metastasis-associated in colon cancer protein 1) [116]. Another typical signaling pathway implicated in EMT is the TGF-β-related pathway. Studies have found that reducing the expression of the transcription factor Oct4 in MCF7 breast cancer cells upregulates ORAI1 expression, leading to TGF-β-stimulated EMT and promoting cell migration and invasion [130]. ORAI3 is also required for TGF-β-dependent Snai1 transcription, a key transcription factor upregulated during EMT. Additionally, hypoxia-induced cell migration and invasion in triple-negative breast cancer cells and colon cancer cells have been linked to the Notch1/ORAI1/SOCE/NFAT4 pathway [131,132]. Conversely, ORAI1 knockdown reduces the turnover rate of BC cell focal adhesions, even though it can be rescued by the small GTPases Ras and Rac [125]. 

The expression of ORAI1 is upregulated in glioblastoma, where it enhances tumor invasiveness and promotes migration by regulating adhesion transition [133]. Interestingly, ORAI1 silencing in a zebrafish nasopharyngeal carcinoma metastasis model reduced cell exudation and inhibited cancer cell adhesion, thus leading to delayed attachment to the extracellular matrix (ECM) surface. In colorectal cancer, higher ORAI1 expression is associated with a more advanced clinical stage, increased metastasis incidence, and shorter overall survival. Kang et al. investigated ORAI1 expression in two colorectal cancer cell lines with differing metastatic potential, SW480 and SW620 cells, and observed significantly higher ORAI1 expression in SW620 cells, which exhibited greater EMT characteristics [134]. Silencing ORAI1 suppressed the EMT of SW620 cells. Under additional TGF-β1 simulation in SW480 cells, there was a notable increase in cell migration along with loss of E-cadherin, elevated N-cadherin and vimentin protein levels, and induction of ORAI1 expression.

### 3.3. ORAI in Tumor Angiogenesis and Metastasis

Efficient blood supply is critical for the survival and proliferation of cancer cells, especially for solid tumors. The newborn blood vessels also provide paths for cancer cells to travel to distant sites. Tumor angiogenesis involves interactions between cells and cells/matrix, activation of receptors, and regulation of angiogenesis factors. The release of vascular endothelial growth factor (VEGF) promotes vessel endothelial proliferation. For example, Epstein–Barr virus (EBV) infection regulates VEGF secretion through SOCE to promote tumor angiogenesis [135]. Hypoxia also induces angiogenesis. In colon cancer and triple-negative breast cancer, hypoxia upregulated ORAI1 by the Notch1 pathway [131]. This is consistent with the findings that Notch1 signaling pathways activate nuclear factor kappa-light-chain-enhancer of activated B cells (NF–κB) and subsequentially regulate ORAI1 expression [136,137]. ORAI1 upregulation activates the nuclear factor of activated T cells, NFAT4, contributing to hypoxia-induced invasion and angiogenesis.

Liu et al. found ORAI2, but not ORAI1 or ORAI3, to be involved in peritoneal metastasis of gastric cancer cells [138]. They found that the expression of NSUN2 (NOP2/Sun RNA Methyltransferase 2), an RNA methyltransferase introducing 5-methylcytosine into tRNAs, mRNAs, and noncoding RNAs, was significantly upregulated in peritoneal metastasis. NSUN2 regulates ORAI2 mRNA stability, then promotes ORAI2 expression and further peritoneal metastasis and colonization of gastric cancers. The whole pathway is identified as the AMPK/E2F1/NSUN2/ORAI2 pathway.

### 3.4. ORAI and Carcinoma Immunity

Immunotherapy stands at the forefront of cancer treatment, with checkpoint inhibitors like CTLA4 (cytotoxic T lymphocyte antigen-4), PD–1 (programmed death-1), and PD–L1 (programmed cell death ligand 1) demonstrating significant efficacy [139,140,141]. Among these, PD–L1 is a pivotal checkpoint molecule that binds to the PD–1 receptor on immune cells, orchestrating an immunosuppressive response critical for immune homeostasis, cancer cell survival, and tissue integrity. Exosome PD–L1 and major histocompatibility complex (MHC) molecules are found to be typical messengers transmitting the immune signal from producer cells to receptor cells in an autocrine or paracrine form [142]. Chen et al. showed that the generation of exosome PD–L1 occurred in a Ca^2+^-dependent manner in a non-small-cell lung cancer mouse model [143]. Knockdown of the gene coding ORAI1 reduced calcium entry and release of small extracellular vesicles (sEVs) without affecting cell proliferation. The total expression of PD–L1 remained the same at the whole-cell level when ORAI1 was knocked down but decreased at the exosome level. The activity of melanophilin and synaptotagmin-like protein 2, important proteins for correct localization of secretory organelles within cancer cells and their transport to sites of exocytosis, were inhibited as well.

## 4. Conclusions

Calcium entry mediated by the ORAI channel is critical for numerous cellular activities, from cell proliferation, energy generation, and gene transcription to procedural cell death. As one of the important components in the CRAC channel, ORAI not only participates in cellular processes but also induces diseases when its function is dysregulated. Typically, gain-of-function mutations cause supercharged calcium entry from the extracellular space and subsequentially induce myopathy [33], whereas loss-of-function mutations result in immunodeficiency [46]. In addition to mutations, post-translational modifications [50], small-molecule inhibitors, and anti-ORAI antibodies [86] can also regulate the function of ORAI. Currently, small-molecule inhibitors are widely applied in both experimental research and clinical trials targeting ORAI to treat pancreatitis [73], immunodeficiency diseases, and other disorders. There are over 10 types of small-molecule inhibitors for ORAI channels [144], but none of them are specific, limiting their clinical applications due to off-target effects. Moreover, there is a lack of effective antibodies to block ORAI channels. Further exploration is needed to develop antibodies that specifically target the ORAI protein, ranging from monoclonal antibodies and single-chain variable fragment (scFv) antibodies to nanobodies.

ORAI channels are found to be overexpressed in various cancers. Notably, the overexpression of ORAI3, rather than ORAI1, plays a predominant role in prostate cancer and breast cancer [105]. The increased calcium entry resulting from the overexpression of ORAI channels promotes cancer cell proliferation, invasion, metastasis [2], tumor angiogenesis, and immune depression. With the development of new inhibitors and antibodies, there is promising potential for treating cancers by targeting ORAI channels in clinical settings.

## Figures and Tables

**Table 1:** Mutations of hORAI1 associated with diseases.

Regions	Mutations	Effects on ORAI	Related Diseases	Ref.
TM1	A88SfsX25	Loss-of-function	Neutropenia and thrombocytopenia, congenital muscular hypotonia, and encephalopathy	[29,30]
	R91W	Loss-of-function	SCID and hypocalcified amelogenesis imperfecta	[13,31]
	S97C	Gain-of-function	TAM and congenital miosis	[32]
	G98S	Gain-of-function	TAM	[33]
	G98R	Loss-of-function	CID, autoimmunity, ectodermal dysplasia with anhidrosis, and muscular dysplasia	[34]
	A103E/L194P	Loss-of-function	Congenital muscular hypotonia, eczema, and neovascularization of cornea	[29,35]
Loop1	V107M	Gain-of-function	TAM	[33,36]
TM2	C126R	Loss-of-function	CID	[37]
	L135R	Loss-of-function	CID	[37]
	A137V	Gain-of-function	Colorectal adenocarcinoma	[38]
	L138F	Gain-of-function	TAM	[27,39]
	M139V	Gain-of-function	Stomach carcinoma	[40]
Loop2	S159L	Gain-of-function	Uterine carcinoma	[41]
	H165PfsX1	Loss-of-function	CID	[42]
TM3	T184M	Gain-of-function	TAM	[36]
	V181SfsX8	Loss-of-function	CID, autoimmunity, ectodermal dysplasia with anhidrosis, and muscular dysplasia	[34]
	L194P	Loss-of-function	CID, autoimmunity, ectodermal dysplasia with anhidrosis, and muscular dysplasia	[34]
Loop3	N233A	Loss-of-function	SCID	[43]
TM4	P245L	Gain-of-function	TAM	[36,44]
	G247S	Gain-of-function	Neck carcinoma	[45]

TM: transmembrane region; Loop1: helix between TM1 and TM2; Loop2: helix between TM2 and TM3; Loop3: helix between TM3 and TM4; SCID: severe combined immunodeficiency; CID: combined immunodeficiency; TAM: tubular aggregate myopathy.
